# Evaluating Material Design Principles for Calcium-Ion Mobility in Intercalation Cathodes

**DOI:** 10.1021/acs.chemmater.4c02927

**Published:** 2024-12-30

**Authors:** Jiyoon Kim, Dogancan Sari, Qian Chen, Gerbrand Ceder, Kristin A. Persson

**Affiliations:** † Department of Materials Science and Engineering, University of California, Berkeley, California 94704, United States; ‡ Materials Sciences Division, Lawrence Berkeley National Laboratory, Berkeley, California 94720, United States

## Abstract

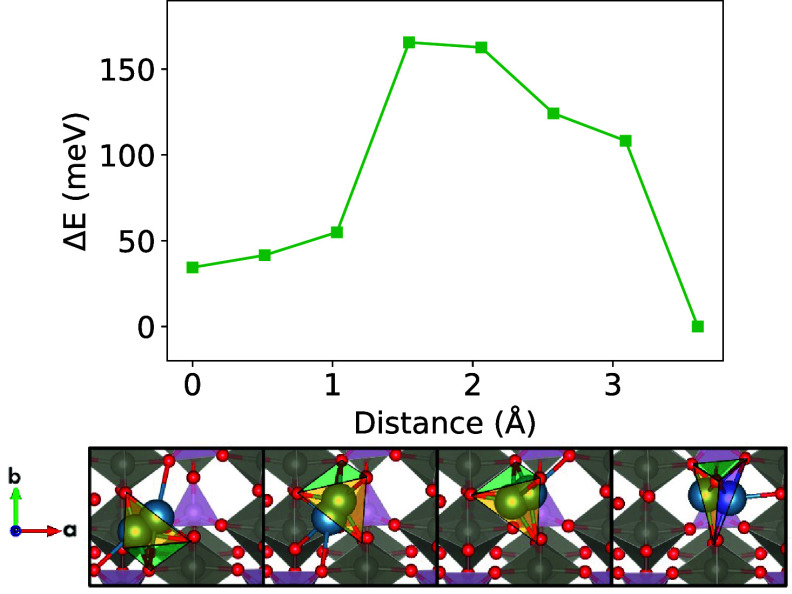

Multivalent-ion batteries offer an alternative to Li-based technologies, with the potential for greater sustainability, improved safety, and higher energy density, primarily due to their rechargeable system featuring a passivating metal anode. Although a system based on the Ca^2+^/Ca couple is particularly attractive given the low electrochemical plating potential of Ca^2+^, the remaining challenge for a viable rechargeable Ca battery is to identify Ca cathodes with fast ion transport. In this work, a high-throughput computational pipeline is adapted to (1) discover novel Ca cathodes in a largely unexplored space of “empty intercalation hosts” and (2) develop material design rules for Ca-ion mobility. One candidate from the screening, W_2_O_3_(PO_4_)_2_, is confirmed to have a low Nudged Elastic Band (NEB) barrier of 168 meV within a one-dimensional (1D) ion percolation topology. This candidate is subsequently synthesized and electrochemically tested, achieving reversible Ca cycling with a capacity of 25 mA h/g. To further accelerate the screening for promising Ca intercalation electrodes, machine learning (ML) Random Forest (RF) and Extreme Gradient Boosting (XGB) classification models are created with local environment descriptors based on a large, structurally and chemically diverse dataset of minimum energy pathways, spanning over 5,000 density functional theory (DFT) site energy calculations. Accuracies of 92% are achieved, material design metrics are quantified, ML force-fields are leveraged in an accelerated iteration of the screening, and a total of 27 novel Ca cathode materials are highlighted for further investigation.

## Introduction

The escalating levels of greenhouse gases have prompted a shift away from fossil fuels;^1^ however, replacement strategies involving intermittent renewable energy production require the parallel implementation of increased energy storage.^2,3^ Today, this demand is almost exclusively met by Li-ion batteries (LIBs), but concerns are raised over the resource scarcity and environmental impact of mining metals (namely Co, Ni and Li) associated with this technology. While Co resources remain insufficient, Ni and Li reserves are also projected to fall short.^4,5^ Higher energy densities, improved safety, and longer cycle life may be achieved with multivalent-ions, because there is an exchange of multiple electrons per charge carrier as well as improved metal plating compared to Li^+^,^6,7^ paving the way toward to a metal anode battery. Calcium batteries are potentially advantageous due to the elemental abundance, nontoxicity, high melting point, and ideal standard reduction potential of Ca^2+^ (−2.87 V vs SHE for Ca^2+^ vs −3.04 V vs SHE for Li^+^), which may allow for higher operating voltages and energy densities.^8,9^ A number of Ca cathode materials have been investigated in the past few years: Na superionic conductor (NASICON) NaV_2_(PO_4_)_3_ with 70 mA h/g at 3.2 V,^10,11^ Na_0.5_VPO_4.8_F_0.7_ with 75 mA h/g at 3.2 V,^12^ NASICON K_3_V_2_(PO_4_)_3_/C with 102 mA h/g at 3.74 V,^13^ and K_*x*∼0_VPO_4_F with 75 mA h/g at 3.85 V.^14^ However, the limited capacity retention of these materials under ambient conditions prompts the need for further advances in Ca-ion battery research. Specifically, we highlight the strong electrostatic interactions between a divalent ion such as Ca^2+^ and the host lattice, which often results in sluggish kinetics and limits the battery’s rate capability.^15^

Past work on Ca battery materials includes screening methods that have been developed using Density Functional Theory (DFT) across spinel, perovskite, and layered structures spanning many chemistries.^16−21^ These efforts focused on compounds with known crystallographic sites for the mobile species, either with existing Ca^2+^ atoms in the structure or via substitution with another working ion. However, cathode materials that are synthesized without the species of interest, here denoted as “empty intercalation hosts”, have largely been unexplored. Examples of such empty intercalation hosts for Li cathodes are graphite and V_2_O_5_. Given that most successful Ca cathodes were originally synthesized without Ca^2+^ (e.g., NASICON NaV_2_(PO_4_)_3_,^10,11^ Na_0.5_VPO_4.8_F_0.7_,^12^ NASICON K_3_V_2_(PO_4_)_3_/C,^13^ K_*x*∼0_VPO_4_F^14^) and material design rules suggest that multivalent-ions in oxides and chalcogenides may be deeply bound in their native crystallographic sites,^22^ there is a strong motivation to explore this larger search space of empty intercalation host structures.

In addition, it is widely recognized that the requirement of fast ionic diffusion in an empty intercalation host structure presents a challenge in identifying competitive Ca cathode candidates. Few experimental examples of fast-rate multivalent cathodes exist,^6,23^ which prompts the use of DFT and high-throughput screening strategies. However, evaluating migration barriers using DFT with methods such as the Nudged Elastic Band (NEB) is time and resource-intensive even with large computational resources. It is therefore of interest to elucidate material design principles to understand the role of chemical and structural features on ion mobility and thus allow for accelerated cathode discovery. Although Ca-ion mobility has been studied in select systems (e.g., spinel Mn_2_O_4_, olivine FePO_4_, layered NiO_2_, orthorhombic δ-V_2_O_5_^22^) and design rules have been rationalized for specific structures with good ion mobility (e.g., MgMo_3_(PO_4_)_3_O,^24^ Mg_3_Bi_2_,^25^ Mg and Ca zircons^26,27^), they have not been quantitatively evaluated at scale, across many structure types, and within a broader chemical search space to predict migration barriers in intercalation electrodes. In this work, we evaluate the mobility of Ca^2+^ in empty intercalation host candidates identified from an adapted high-throughput computational pipeline^28^ to understand motifs favorable for ion diffusion and to identify promising Ca cathodes. A large multivalent-ion mobility data set is generated with DFT, material design metrics are evaluated for Ca-ion mobility, machine learning (ML) models are produced using local environment descriptors, and ML force-fields are applied to quickly identify promising Ca cathodes.

## Methods

### Screening Methodology

We evaluate Ca cathode properties such as their chemistry, stability, electrode performance, and ion mobility with screening tools of increasing computational cost. From 126,335 compounds in the 2022 version of the Materials Project database,^29^ we identify 7,681 empty intercalation host materials that meet the following criteria: (1) has an energy above the hull less than 0.2 eV/atom to promote synthesizability,^30^ (2) contains a redox-active metal that can access lower oxidation states upon calciation (Ti^4+^, V^4–5+^, Cr^4–6+^, Mn^3–7+^, Fe^3–6+^, Co^3–5+^, Ni^3–4+^, Cu^2–4+^, Nb^5+^, Mo^4–6+^, Ru^5–8+^, Ag^2–3+^, W^6+^, Re^7+^, Sb^5+^, Bi^4–5+^), (3) has oxygen or sulfur anions, (4) is free of radioactive elements, and (5) no additional intercalating ions (H^+^, Li^+^, Na^+^, Mg^2+^, K^+^, Ca^2+^, Cu^1–3+^, Rb^+^, Ag^1–3+^, Cs^+^) are present to simplify the evaluation of ion migration and mobility. Next, we determine potential intercalation sites using an insertion algorithm, where Ca^2+^ is inserted in sites with local electronic charge density minima because this has been found to correlate with viable intercalation sites.^31^ For each cathode candidate, the most stable, DFT-relaxed structure with topotactic ion insertion, in which the host material does not undergo significant structural changes upon calciation, is selected for further investigation in the pipeline. To identify a topotactic ion insertion, we verify positive structure matching between the empty host and calciated material using pymatgen^32^ and the following tolerance parameters: fractional length “ltol” of 0.4, site position “stol” of 0.6, and relative angles “angletol” of 10°. Of the 7,681 host materials identified in the phase stability and chemistry phase of the screening, we prioritize the 2,238 candidates that are present in the Inorganic Crystal Structure Database (ICSD) due to their lower energy above the hull and hence higher chance of synthesizability. We apply the insertion algorithm to these 2,238 compounds and identify 1,732 candidates with successful topotactic ion insertions. Ca cathode candidates that satisfy the following conditions are subsequently evaluated for Ca-ion mobility: (1) discharged state energy above the hull lower than 0.2 eV/atom, (2) intercalation voltage between 0.5 and 4.0 V, (3) conversion voltage no greater than 1 V above the intercalation voltage, and (4) volume change upon intercalation less than 20%. Criterion (1) is applied, because the literature reports energy above the hull values up to 0.2 eV/atom for the 90^*th*^ percentile of metastable, synthesizable polymorphs,^30,33^ and it avoids screening out materials that could be viable with lower levels of Ca intercalation. A periodic graph known as a “migration graph” is constructed with symmetrically-equivalent intercalation sites to identify diffusion hops between metastable Ca sites in candidate materials.^34^ Materials without percolating pathways are deprioritized, resulting in 242 candidates for barrier evaluation. We apply an efficient and robust alternative to NEB known as Approximate NEB (ApproxNEB)^35^ to these 242 materials and calculate their migration energies for Ca^2+^ in the dilute lattice limit. Energy barriers are mapped back onto their respective migration graphs to locate the lowest energy, percolating pathway for each candidate per Dijkstra’s algorithm.^34^ It is worth noting that some ApproxNEB calculations do not converge electronically, which is usually associated with a very high barrier (>1 eV). In our work, 612 of 816 DFT calculations converged, producing ion mobility data for 213 candidates. This ApproxNEB dataset is used for local environment analysis and machine learning. Chosen numerical and convergence parameters for these calculations are identical to those in previously published work.^27^

### Electrodes Cost Function

To enable easier comparison across candidate cathode materials of varying chemistry and structure types, we construct cost functions for the voltage, phase stability, and volume change. These functions are applied to 1,732 intercalation electrodes with topotactic Ca-ion insertion. Intercalation voltages are calculated using the total energies of the host and calciated structures, which are derived from first-principles.^36^ Conversion voltages are theoretically evaluated in a similar manner using pymatgen^32^ and available Materials Project phase diagram data.^29^ The thermodynamic stability of the compounds is estimated using the energy above the convex hull, based on the MP2020 Compatibility Scheme^37^ and generated phase diagrams.^29^ The convex hull is comprised of the most stable phases at 0 K based on DFT in a given chemical space.^38,39^ For each cathode candidate, the cost function is cast as a sum of parabolic functions, where *C*_*total*_ is the total cost function value, *p*_*i*_ is the value of the electrode property *i* of interest (e.g., intercalation voltage), *p*_*i*,0_ is the value of the transition point for a given cost function parabola, and *p*_*i,1*_ is the value of the lower or upper bound at which the cost equals 1 (eq 1). A cost function value exceeding 1 for a given electrode property is considered suboptimal for experimental testing. Plots illustrating the cost as a function of each parameter are provided in Figure S1.
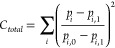
1

### Random Forest and Extreme Gradient Boosting Models

We create Random Forest (RF) and Extreme Gradient Boosting (XGB) classification models using the Python package scikit-learn.^40^ The following settings are implemented: 200 estimators for both models and a maximum tree depth of five for the XGB model. All other hyperparameters are set to their default values. Features are selected according to the methodology outlined by Hapfelmeier et al.^41^ 5-fold cross-validation is applied to test the performance of the models, and features are ranked based on their Gini importance. The ten features with the greatest Gini importance values are implemented in the final models.

### Experimental Synthesis

W_2_O_3_(PO_4_)_2_ is created via solid-state synthesis techniques using stoichiometric ratios of WO_3_ (99.99%) and P_2_O_5_ (99%) precursors from Sigma-Aldrich. The precursors are mixed by ball milling at 300 rpm for 4 h with Zirconia balls to a powder weight ratio of 10:1. Pellets are formed from this mixture prior to heat treatment by applying a 200 MPa uniaxial pressure, are heated to 700 °C under air, and then maintained at this temperature for 7 days.

### Electrochemical Testing

The active material, carbon black (Timcal, SUPER C65) and polytetrafluoroethylene (PTFE, DuPont Teflon 8A) are mixed in a glovebox at a weight ratio of 7:2:1 to prepare the cathode films. Activated charcoal carbon (AC, Sigma), carbon black, and PTFE are combined at a weight ratio of 8:1:1 in a glovebox to create the anodes. The mixtures are rolled to form thin film cathodes and anodes. The coin cells are prepared with these cathode and anode thin films with a loading density of 3 mg/cm^2^ and 20 mg/cm^2^ for the cathode and anode, respectively.

The electrolyte is prepared by drying calcium(II) bis(trifluoromethanesulfonyl)imide (Ca(TFSI)_2_, 99.5%, Solvionic) salt at 170 °C overnight in an Ar-filled glovebox. The dried salt is used to form 0.5 M Ca(TFSI)_2_ in diglyme (99.5%, Sigma-Aldrich). The electrolyte and its components are always kept inside a glovebox.

Coin cells are assembled with the electrolyte, cathode and anode thin films, and separators (Whatman glass microfiber filter). Galvanostatic cycling tests are performed at 50 °C with an Arbin battery tester. The tests are conducted at a current density of 2 mA/g, and ex-situ samples are collected after washing the cathode thin films with diglyme in an Ar-filled glovebox.

### X-ray Diffraction Characterization

The phase identification of the synthesized samples and structural changes in the cathodes are observed with X-ray diffraction (XRD) using a Rigaku MiniFlex 600 diffractometer with Cu Kα radiation (λ = 1.54178 Å) in the 2θ range of 10° to 60°.

## Results and Discussion

### Analysis of Ranked Electrodes

Cost functions are created for the following parameters: intercalation voltage, difference between the conversion and intercalation voltage, phase stability for the host and calciated structure, and volume change upon Ca intercalation. The cost function value parabolically increases when the electrode property *i* of interest strays from *p*_*i,*0_, which is the metric set to indicate good viability for experimental testing. Candidates with cost function values ≤1 for each electrode property are considered for further analysis. The acceptable intercalation voltage range is based on experimentally accessible voltages and the stability window of the electrolyte. While a cathode with a conversion voltage higher than its intercalation voltage can be kinetically stabilized and allow for the reversible intercalation of the working ion (e.g., Mg in spinel Ti_2_S_4_^42^), a stronger thermodynamic driving force for conversion is typically undesirable. A maximum voltage difference of *V_conversion_* – *V*_*intercalation*_ = 0.5 V is referenced to an experimentally verified conversion reaction threshold,^43^ with a 25% buffer due to potential overestimations of the conversion voltage in our theoretical work. A cutoff of 100 meV/atom for the energy above the hull is implemented per benchmarking analysis on synthesizable, crystalline inorganic materials.^33^ Lastly, an upper limit of 15% volume change is set based on typical volume changes for commercially available layered anode materials such as graphite,^44^ while also acknowledging that Ca intercalation is likely to incur higher volume changes per inserted ion compared to Li^+^. We apply these cost functions to the 1,732 candidates with topotactic Ca intercalation.

A demonstration of electrode ranking with these cost functions is tabulated in Table S1. The candidates in each tier are representative of the lowest, median, and highest total cost function value within each group. The first 3 candidates have electrode properties that would prioritize them for ion mobility evaluation and potentially experimental testing. These tier 1 electrodes are defined to have a cost function value <1 in each of the categories. The next group of 3 materials have either one or more electrode properties that could challenge their viability, and the last 3 are deprioritized. Roughly 6% of the 1,732 electrodes fall under the tier 1 category, whereas 59% exhibit too low or too high intercalation voltages, 88% favor conversion reactions, 49% have poor phase stability, and 24% experience large volume changes upon calciation among the tier 2 and 3 candidates. Interestingly, only 12% of candidates exhibit a reasonably low thermodynamic driving force for conversion, which indicates that conversion reactions are highly competitive. Notably, this useful screening criteria as well as the volume change upon intercalation were not considered in the previous Mg cathode pipeline.^28^

The electrode properties for the ten tier 1 candidates with the lowest total cost function value and highest gravimetric capacities are displayed in Table 1. Some of these materials have been explored as intercalation electrodes in the literature (e.g., Li_*x*_VP_2_O_7_,^45^ Mg_*x*_MoOPO_4_,^46^ Ca_*x*_VO_2_,^47^ Mg–Li hybrid VO_2_ batteries,^48^ [Ca,Zn]_*x*_V_2_O_5_,^49,50^ [Ca,Mg]_*x*_ABO_4_ zircons^26,27^) either because they have an existing discharged state or belong to a well-established family of cathode compounds. The “rediscovery” of such cathode materials that have previously shown viability in electrochemical testing demonstrates the effectiveness of the cost functions and of the overall screening pipeline for multivalent cathode discovery. Equally encouraging, there are a number of candidates that have not been previously studied as Ca cathodes or as cathodes in general (e.g., FeMoClO_4_, SrFe_2_(P_2_O_7_)_2_, Cu_2_SO_5_).

**Table 1:** Top Ten Ca Cathodes Tabulated in Ascending Order of Total Cost Function Value, with the Highest-Performing Candidate Listed First^a^^b^^c^

Composition MP-ID	Prototype Structure	Intercalation Voltage (V)	Charged Stability (meV/Atom)	Discharged Stability (meV/Atom)	ΔVolume (%)	Gravimetric Capacity (mA h/g)
Ca0–0.5FeMoClO_4_	-	3.1	17	12	6	99
mp-23123						
Ca0–0.5VP_2_O_7_	LiVP_2_O_7_	3.4	2	0	6	109
mp-25294						
Ca0–0.5MoOPO_4_	-	3.1	2	16	10	118
mp-1104182						
Ca0–0.25VO_2_	TiO_2_	2.5	29	54	5	144
mp-541404						
Ca0–1SrFe_2_(P_2_O_7_)_2_	-	3.0	0	30	7	91
mp-556067						
Ca0–0.5V_2_O_5_	-	3.2	0	54	2	133
mp-754670						
Ca0–0.5V_2_O_5_	-	3.2	0	54	4	133
mp-25279						
Ca0–0.5Cu_2_SO_5_	-	3.1	9	59	11	103
mp-4386						
Ca0–0.5EuVO_4_	zircon	2.2	0	42	15	93
mp-22796						
Ca0–0.5V_2_O_5_	-	3.2	25	75	14	133
mp-510568						

a The MP-ID is the Materials Project identifier.^51^

b The cathode prototype structure references either the most known material in the same structural family or their group name.

c Voltages are with respect to Ca/Ca^2+^.

### Candidates Explored in Depth

Candidates with tier 1 and 2 electrode properties that have not yet been studied as Ca cathodes in the literature are evaluated for ion mobility. The electrode properties and ApproxNEB (or NEB if available) barriers for candidates with percolating migration pathways are tabulated (Table 2). Notably, these materials do not have known cathode prototype structures, e.g., we have not found any previous examination of an intercalation cathode material within the same structural family. FeMoClO_4_ and FeWClO_4_ belong to the same structural group, have similar migration energy barriers as well as intercalation voltages, but FeMoClO_4_ has slightly superior phase stability and gravimetric capacity compared to the tungsten-based material. ϵ-VOPO_4_ was recently identified as a Mg cathode^52^ through the same screening approach^28^ and was validated experimentally, but here the δ polymorph is identified as a possible Ca cathode. Although several VOPO_4_ polymorphs have been thoroughly studied in Li and Na-ion batteries,^53,54^ only the α phase has been evaluated as a water-activated Ca cathode.^55^ Zhao et al. were able to retain 91 mA h/g for an impressive 1000 cycles at 1.46 V with δ-VOPO_4_ in aqueous Zn-ion batteries,^56^ which motivates further inquiry into δ-VOPO_4_ with Ca^2+^ given its higher predicted voltage of 3.0 V. All but two candidates, structurally similar FeMoClO_4_ and FeWClO_4_, exhibit one-dimensional (1D) diffusion channels. Although the gravimetric capacity of some of these candidates are low (Table 2), several of these prospective cathode materials can theoretically achieve higher capacities with multiple Ca insertions (e.g., Ca_0.5_FeMoClO_4_, Ca_0.13_VOPO_4_) since our screening pipeline performs a limited evaluation of their electrode properties based on one intercalation event. We further computationally investigate two candidates, W_2_O_3_(PO_4_)_2_ and NbS_3_, due to their low migration barriers for Ca^2+^ ions.

**Table 2:** Electrode Properties and Ca-Ion Mobility Data for Candidates with Tier 1 or 2 Cost Function Values and Percolating Pathways with Migration Barriers ≤650 meV for Reasonable Cycling^22^^a^^b^^c^^d^

Composition MP-ID	Intercalation Voltage (V)	Charged Stability (meV/Atom)	Discharged Stability (meV/Atom)	ΔVolume (%)	Gravimetric Capacity (mA h/g)	Migration Barrier (meV)	Diffusion Dimension
Ca0–1.5W_2_O_3_(PO_4_)_2_	2.5	0	99	10	125	168*	1
mp-19522							
Ca0–0.5W(S_4_Cl_3_)_2_	2.4	1	42	3	40	193	1
mp-28575							
Ca0–0.17NbS_3_	1.3	0	61	3	46	333*	1
mp-1190583							
Ca0–0.5FeMoClO_4_	3.1	17	12	6	99	432	2
mp-23123							
Ca0–0.5FeWClO_4_	3.1	71	70	7	75	464	2
mp-556603							
Ca0–0.17FeAsO_4_	3.2	0	23	1	44	527	1
mp-19398							
Ca0–0.25VN_3_O_10_	3.2	0	19	3	51	571	1
mp-1205113							
Ca0–0.13VOPO_4_	3.0	0	19	6	40	588	1
mp-554181							
Ca0–0.25WOF_4_	3.8	10	0	10	47	646	1
mp-540636							

a The MP-ID is the Materials Project identifier.^51^

b Voltages are with respect to Ca/Ca^2+^.

c The charged and discharged stability describe the energy above the hull values for the host and calciated structures, respectively.

d The migration barriers are calculated with ApproxNEB or NEB* in the dilute lattice limit.

Orthorhombic Ca_0–1.5_W_2_O_3_(PO_4_)_2_ has a theoretical intercalation voltage of 2.5 V, a volume change of 10%, an energy above the hull of 99 meV/atom, and a gravimetric capacity of 125 mA h/g (Table 2). Electrochemical cycling with Li^+^ and Na^+^ has been performed on the monoclinic polymorph of Ca_0–1.5_W_2_O_3_(PO_4_)_2_ due to its structural similarity to previously studied polyanionic frameworks for LIBs (e.g., Fe_2_(WO_4_)_3_ and Fe_2_(MoO_4_)_3_),^57^ with Li^+^ showing greater success as the working ion. Although Maddukuri et al. were unable to fully deintercalate Li^+^ in the initial cycles, they maintained a capacity of 65 mA h/g for 25 cycles with a Coulombic efficiency of 99%. Since the monoclinic and orthorhombic polymorphs exhibit similar stability metrics (formation energy of −2.378 eV/atom and −2.385 eV/atom, respectively) and both consist of corner-sharing WO_6_ octahedra and PO_4_ tetrahedra albeit with slightly different tilt angles, their electrochemical performance is expected to be similar. We perform Ca, Li, and Na intercalation on the orthorhombic W_2_O_3_(PO_4_)_2_ structure due to the lack of computational data in the literature. Although the discharged structure is the least stable with Ca^2+^, a concentration of 1.5 Ca^2+^ per formula unit is possible without reaching the redox limit of W^4+^ (Figure S2). The theoretical voltages with Ca^2+^ vs Li^+^ are comparable, whereas the intercalation voltage with Na^+^ is almost 1 V lower. As a result, the sodiated cathode strongly favors conversion by 2.7 V. This may explain the poor electrochemical performance of monoclinic Na_*x*_W_2_O_3_(PO_4_)_2_, highlighting Ca^2+^ as a more favorable ion to study.

**Figure 1:**
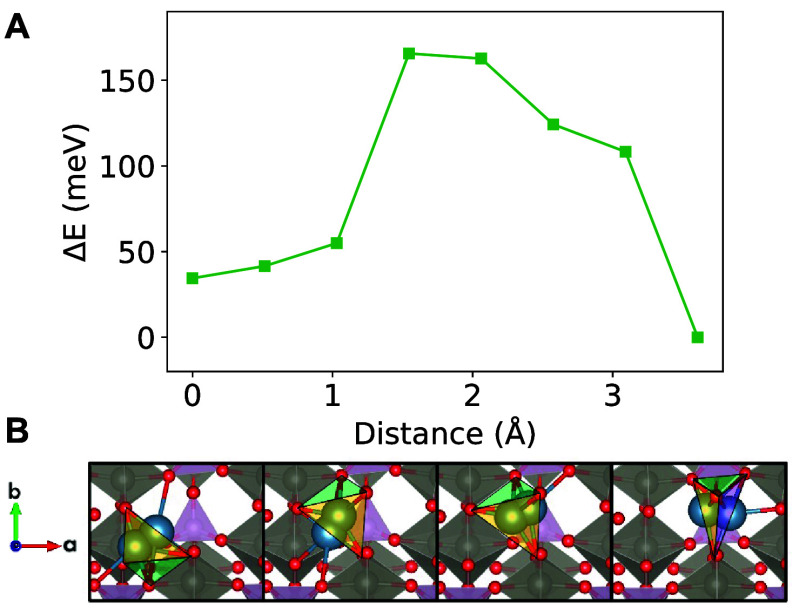
(A) NEB energy profile for Ca_*x*_W_2_O_3_(PO_4_)_2_ in the dilute lattice limit (*x* ≤ 0.125 with 1 Ca-ion per supercell structure). (B) The tetrahedral overlap of nearest-neighbor atoms around Ca^2+^ (in blue) along the migration pathway is illustrated below.

Climbing image NEB calculations confirm a remarkably low migration barrier of 168 meV (Figure 1A). The fast Ca-ion diffusion in W_2_O_3_(PO_4_)_2_ may be attributed to the low coordination change of Ca^2+^ as it migrates (e.g., coordination of 5, 6, 4, 5). Furthermore, the consistent tetrahedral overlap of the nearest-neighbor atoms around Ca^2+^ interstitial sites along the pathway is more conducive to good ion mobility (Figure 1B), in contrast to traveling through a plane of anions which has been correlated with poor diffusion.^28^ Although W(S_4_Cl_3_)_2_ is not evaluated in more depth, it shares the same coordination landscape as W_2_O_3_(PO_4_)_2_ except for a consistent nearest-neighbor overlap of 4 and 5, which may explain the ultralow barriers in both candidates. It is also a 1D diffuser. Since W_2_O_3_(PO_4_)_2_ is the most promising in terms of capacity and migration barrier, Ab Initio Molecular Dynamics (AIMD) simulations are performed to verify the dimensionality of the percolating pathway. This analysis reveals that Ca^2+^ is likely to travel in 1D (Figure S3), which is problematic if impurities or native antisite defects block the diffusion channel.^58^ Despite the slight differences between the AIMD and NEB pathways (most evident within the a-c plane), the activation energy of 159 ± 30 meV calculated from molecular dynamics is consistent with the NEB results (Figure S4).

**Figure 2:**
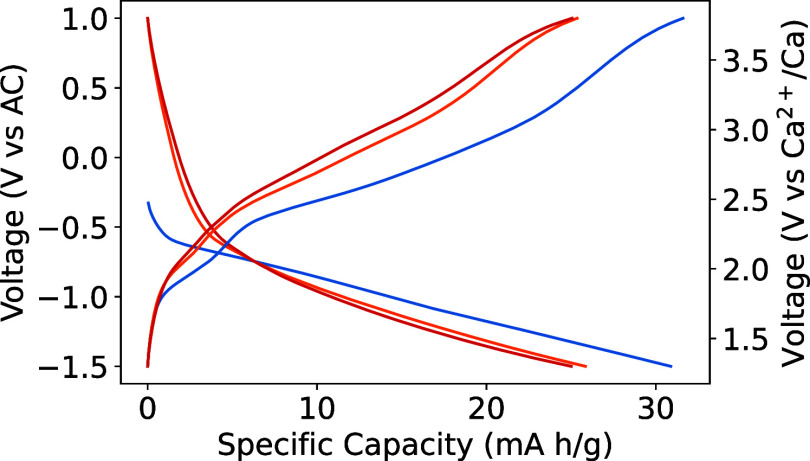
Charge and discharge profiles of W_2_O_3_(PO_4_)_2_ against an AC anode at 50 °C with a current density of 2 mA/g.

We synthesize W_2_O_3_(PO_4_)_2_ with solid-state techniques and use oxide precursors (WO_3_, P_2_O_5_). Details for this synthesis can be found in the Methods section. XRD is performed to characterize the phases present in the cathode material (Figure S5). Extra peaks are observed alongside those corresponding to W_2_O_3_(PO_4_)_2_ across several characterization attempts, and further sintering is found to decrease the relative intensity of these additional peaks, but it is unsuccessful at completely removing them. Although we are unable to define the secondary phases and the quality of the refinement is limited, the dominant phase in the final product appears to be W_2_O_3_(PO_4_)_2_ (Figure S5). The charge and discharge profiles of the coin cells are measured against the AC anode and customized electrolyte 0.5 M Ca(TFSI)_2_ in diglyme at 50 °C with a current density of 2 mA/g. This setup and cell components allow measurements in the voltage range of −2 to 1 V vs AC. The compound achieves a specific capacity of 32 mA h/g in the initial cycle and decreases to 25 mA h/g in subsequent cycles (Figure 2). The average experimental voltage of 2.44 V vs Ca/Ca^2+^ is consistent with the theoretically predicted voltage of 2.50 V. It is possible that defects in the 1D diffusion channels are limiting the extent of Ca intercalation in W_2_O_3_(PO_4_)_2_. We observe a drop in specific capacity after the first cycle, as did Maddukuri et al. when they electrochemically tested the monoclinic polymorph with Li.^57^ Although the compound yields a lower capacity than anticipated from computational analysis, it demonstrates the effectiveness of identifying cathode candidates that can electrochemically cycle Ca^2+^ using a high-throughput approach.

Monoclinic Ca_0–0.17_NbS_3_ is predicted to exhibit an intercalation voltage of 1.3 V, a 3% volume change upon calciation, a maximum energy above the hull of 61 meV/atom, and a gravimetric capacity of 46 mA h/g (Table 2). Monoclinic and triclinic [Li,Mg,Zn]_*x*_NbS_3_ have been previously synthesized under high pressure conditions and tested as cathodes.^59,60^ The highest capacity retention was achieved with cycling Li^+^ in the monoclinic phase. Although Yamamoto et al. were able to repeatedly intercalate and extract Li^+^, they could not fully deintercalate Li^+^ and observed irreversible Li accumulation in the cathode over the course of 120 cycles. Since DFT has not been performed to evaluate these materials as cathodes in the literature, their electrode properties and ion mobility are calculated in this work. In particular, we find that Ca intercalation may be limited due to the poor phase stability of the discharged state above 0.17 Ca^2+^ per formula unit (Figure S6A). Of the three working ions examined computationally (Ca^2+^, Li^+^, Mg^2+^), only the magnesiated cathode has a negative intercalation voltage of −0.6 V, which may explain its poor electrochemical performance^60^ (Figure S6B). Although persulfide bonds are present in NbS_3_ and it is possible for S^–^ to be reduced, Bader charge analysis suggests that only Nb^5+^ is redox-active.^61,62^

**Figure 3:**
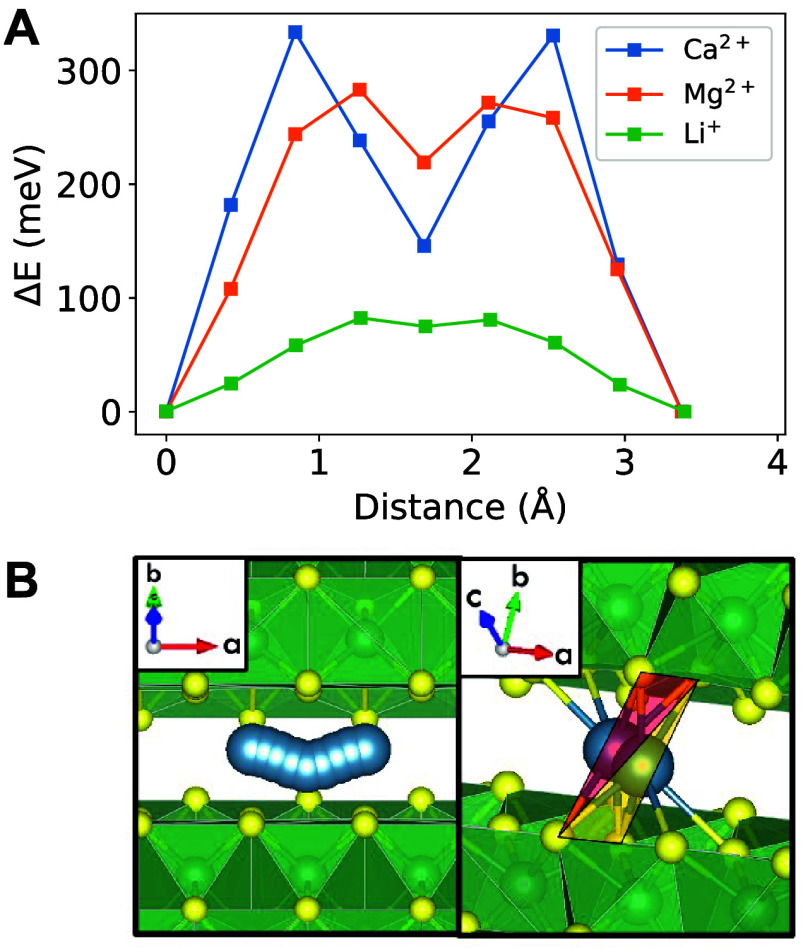
(A) NEB energy profiles for Ca_*x*_NbS_3_, Li_*x*_NbS_3_, and Mg_*x*_NbS_3_ in the dilute lattice limit (*x* ≤ 0.04 with 1 ion per supercell structure). (B) Left: Migration pathway of Ca^2+^ (in blue) between layers in Ca_*x*_NbS_3_ from NEB calculations (*x* ≤ 0.04 with 1 Ca-ion per supercell structure). Right: Ca^2+^ in octahedral sites with a tetrahedral nearest-neighbor overlap in the migration pathway.

NEB calculations are performed with Ca^2+^, Li^+^, Mg^2+^ as the mobile species. Energy barriers of 333, 82, and 283 meV, respectively, are confirmed in the interlayer diffusion pathways (Figure 3A). The flatter energy landscape may be attributed to the reduced site preference of the working ion that results from the octahedral environments with tetrahedral nearest-neighbor overlap (Figure 3B), which has also been identified in the zircon family.^26,27,63^

### Evaluating Material Design Rules

Although the tetrahedral overlap and octahedral overlap by tetrahedra motifs are present in the cathodes above, there are additional factors that influence the overall mobility of Ca^2+^. Past work has identified descriptors that may inform ion mobility in intercalation electrode materials: reduced migration barriers due to coordination preferences of the mobile species,^22^ minimal coordination change of the working ion,^24,25^ larger volumes per anion in the host framework structure,^20^ and distorted, interlocking octahedral polyhedra that overlap by tetrahedra.^26,27^ However, material design rules that influence multivalent-ion mobility have not yet been evaluated at this scale, across many structure types, and within a wide chemical space. Though ML force-fields have become popular and more accurate in predicting properties such as the formation energy (e.g., M3GNet,^64^ CHGNet,^65^ MACE^66^), they tend to flatten and smooth the electrostatic landscape, leading to a systematic underestimation of ionic migration barriers.^66^ Indeed, the high sensitivity of the minimum energy migration path to its local environment poses a challenge to data-driven methodologies. Furthermore, performing DFT calculations is time-consuming and resource intensive, which has limited efforts to uncover design principles for fast ion transport and quantify their influence to accelerate the cathode screening process.

**Figure 4:**
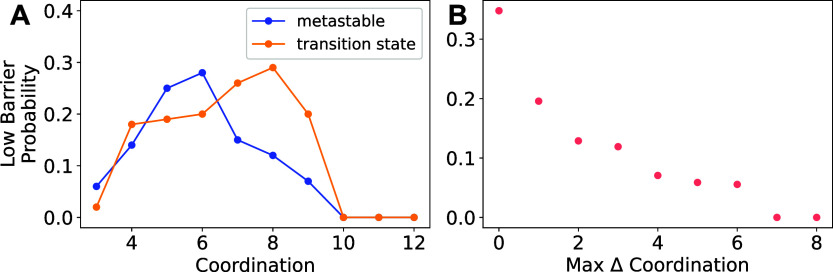
Probability of identifying a low ApproxNEB energy barrier (≤650 meV) given the (A) coordination of Ca^2+^ at the metastable/intercalation site vs the transition state site and (B) maximum coordination change of Ca^2+^ within the pathway.

Upon analyzing 612 symmetrically unique ApproxNEB pathways across 213 compounds, consisting of 5,508 relaxed supercell structures across 182 structure groups and 48 elements, certain structural features are found to affect Ca-ion mobility. We find it is generally true that materials with higher volumes per anion (e.g., O^–^, S^–^) exhibit lower Ca migration barriers (Figure S7). It is possible that materials with low migration energies but higher densities of anions have good electrostatic screening. The volume per S^–^ is on average slightly higher than the volume per O^–^; however, it should be noted that there are 14 times more oxides than sulfides in this data set, which may skew the statistics. Based on a comprehensive coordination study of cations in the ICSD,^67^ Ca^2+^ is known to prefer higher coordination values of 6 to 8. The impact of the coordination landscape on Ca-ion mobility is investigated in all 612 migration pathways and measured with respect to the low barrier probability, which is defined as the ratio of low barrier pathways to the total number of pathways that satisfy a given coordination metric. We observe a higher probability of low barrier pathways when Ca^2+^ has a coordination of 5 or 6 in its metastable site and 7 or 8 in its transition state site (Figure 4A). This supports the hypothesis that the unpreferred coordination in the metastable site flattens the energy landscape, as previously identified in the local environment analysis of Li^+^, Mg^2+^, Zn^2+^, Ca^2+^, and Al^3+^ in Mn_2_O_4_, FePO_4_, NiO_2_, and δ-V_2_O_5_.^22^ Furthermore, a more favorable coordination in the transition state site promotes good ion mobility. In agreement with previous studies,^24,25^ a correlation is observed between more uniform Ca coordination along the migration pathway and lower energy barriers (Figure 4B), which can reduce the site preference for Ca^2+^.

**Figure 5:**
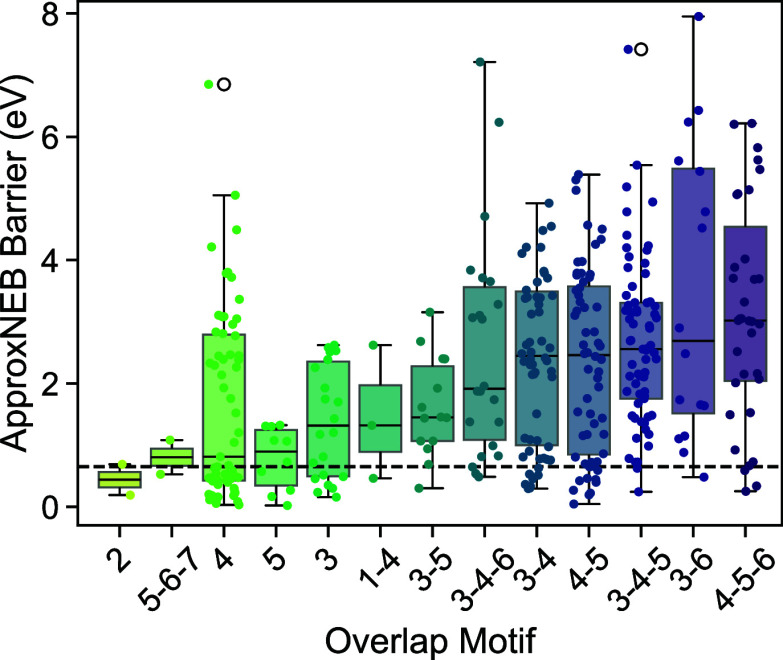
Motif of nearest-neighbor overlap of Ca^2+^ between adjacent image structures in ApproxNEB pathways. Only motifs with at least one barrier ≤650 meV are plotted.

The migration pathway and energies of multivalent-ions in cathode materials are strongly influenced by their local environment, as seen in this work as well as in the literature.^26,27,63^ To investigate this further and gain insight on structural descriptors that affect ion mobility, we characterize the local environments of Ca^2+^ across 612 ApproxNEB pathways spanning 213 compounds. Nearest-neighbor analysis is performed with the crystal-near-neighbor (CrystalNN) algorithm, selected due to its robustness and strong agreement with coordination analysis reported in the literature.^68^ The nearest-neighbors of adjacent Ca^2+^ interstitial sites along a given migration pathway are compared and the number of nearest-neighbors in common, known as the nearest-neighbor overlap, is counted. Given that ApproxNEB is performed with 7 evenly spaced images between migration end point structures of varying path lengths in our data set, the nearest-neighbor overlap is calculated between image structures with interstitial sites that are at most 1.43 A apart. This interimage distance threshold is calculated based on analysis of the tetrahedral nearest-neighbor overlap in 21 zircons, NbS_3_, and W_2_O_3_(PO_4_)_2_. The most common nearest-neighbor overlap motifs, which is defined as the unique set of overlap values within a pathway, are 3–4–5 in 69 pathways, 3–4 in 62 pathways, 4–5 in 60 pathways, and 4 in 60 pathways. The overlap patterns associated with the most pathways with barriers ≤650 meV are 4, 3–4, and 4–5 (Figure 5). Generally, the motifs with at least one low barrier migration pathway consist of overlap numbers close in value (e.g., 3–5, 4–5–6 vs 0–6, 4–9). Pathways with minor nearest-neighbor overlap may exhibit flatter energy landscapes due to the slowly varying local environment and corresponding site energies. For example, ApproxNEB pathways with a consistent overlap of 2 are identified in Mo_8_O_23_ (mp-2669) and BaMo_2_(PO_6_)_2_ (mp-555338) with barriers of 189 and 687 meV, respectively. Analysis of the site energy differences and nearest-neighbor overlap of adjacent image structures within their respective pathway reveals that a majority of low site energy differences is found when tetrahedral nearest-neighbor overlap occurs (Figure S8), suggesting that this motif is favorable for ion mobility. Low barrier pathways in both candidates that are explored further, W_2_O_3_(PO_4_)_2_ and NbS_3_, also exhibit this coordination pattern, and the latter shares a distinct motif of interlocking, distorted octahedra with the zircon family.^26,27,63^ Several working ions (Na^+^, Ca^2+^, Mg^2+^, and Zn^2+^) have been found to have good ion mobility in the zircons, suggesting the importance of investigating this specific motif and the tetrahedral overlap in other systems with different mobile species. All 41 energy barriers for pathways where Ca^2+^ migrates through a plane of anions are greater than 650 meV, which supports previous research on factors that may slow multivalent-ion diffusion.^28^ This finding also correlates with high migration barriers for Ca^2+^ in olivine FePO_4_ and layered NiO_2_, where Ca^2+^ is tetrahedrally coordinated at the saddle point, as opposed to lower barriers in spinel Mn_2_O_4_ because Ca^2+^ migrates through an intermediate octahedral site.^19,22^

We also quantify the influence of the structural features described above on the energy landscape. Additional featurizers from the Python package matminer^69^ are implemented (e.g., ElectronAffinity, ElectronegativityDiff, DensityFeatures, AGNIFingerprints, CrystalNNFingerprint, BondOrientationalParameter) to produce ML models that classify migration energy barriers as insufficient or sufficient for electrochemical cycling (≤650 meV for cycling nanosized particles at a C/2 rate^22^). A classification model is selected over a regression-based algorithm, because pathways with poor ion mobility dominate this data set of 612 ApproxNEB pathway calculations across 213 materials: 88% of barriers are >650 meV and 60% of barriers are >2,000 meV (Figure S9). This would otherwise lead to poor migration energy predictions because regression-based ML models aim to minimize the mean squared error, which would skew toward high migration barrier pathways. A classification-based approach is more effective for this use case, as its algorithm aligns with the objective of this work for migration barrier screening. RF and XGB classification models are trained using feature data generated from the 612 relaxed ApproxNEB pathways. Accuracies of 92.70% and 92.31% are obtained by predicting barrier classifications with the RF and XGB models, respectively. Both perform well, given that acceptable models have an accuracy >70%.

**Figure 6:**
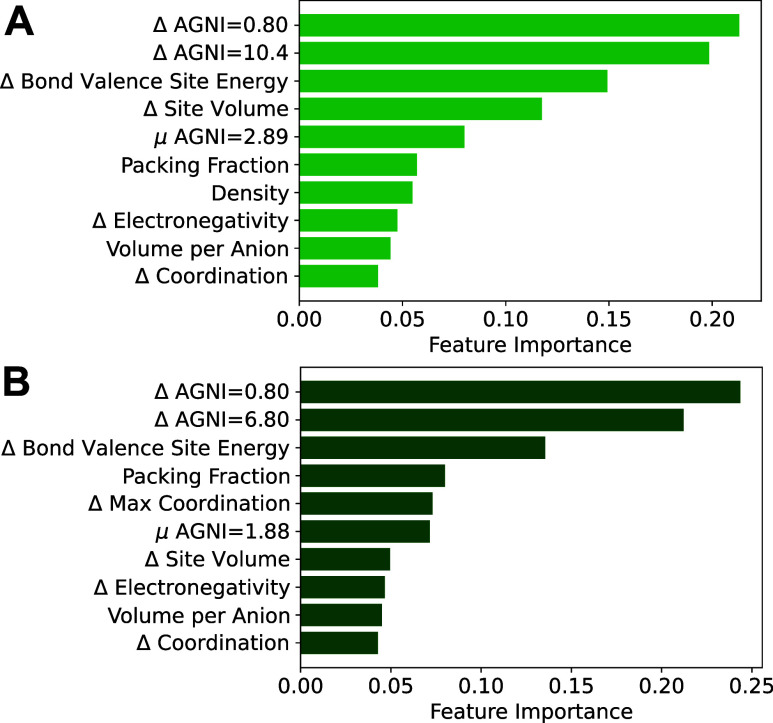
Top 10 features and their Gini importance values in the (A) RF model and (B) XGB model, which are generated with features from relaxed ApproxNEB pathways with a barrier classification threshold of 650 meV.

The features with the greatest Gini importance are largely the same in both models. Relative changes in the Adaptive, Generalizable, and Neighborhood-Informed (AGNI) fingerprint^70^ of Ca^2+^ and in the energy of the bond valence site^71^ along a migration pathway generally carry the most weight (Figure 6). The AGNI fingerprint  for atom *i* along a direction *k* captures information from the radial distribution function within wide and narrow Gaussian windows, when sampling η between 0.8 A and 16 A on a logarithmic grid (eq 2).^70^ The distance between atom *i* and its neighbor *j* that is within a radius of 6.5 A is represented by *r*_*ij*_, and  symbolizes the scalar projection of this distance along the direction *k*. A cutoff distance *R*_*c*_ of 8 A is fed into the cosine damping function, decreasing the influence of atoms far away from atom *i*. As a numeric reference for these fingerprint values, examples of low and high barrier migration pathways are provided (Figure S10). The spacious channel in Tl_2_TeMo_2_(PO_7_)_2_ results in a barrier of 156 meV, whereas the barrier is 9,211 meV when Ca^2+^ squeezes through a trigonal plane of anions in MnO_2_. For η = 0.80, the AGNI fingerprint is 0.00138 and 0.00196, while it is 23.557 and 32.457 when η = 16.0 in Tl_2_TeMo_2_(PO_7_)_2_ and MnO_2_, respectively. The fingerprint values increase by roughly 38–43% as the energy landscape increasingly varies, which explains their high Gini importance in the ML models.
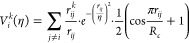
2

In addition, the empirical electrostatic valence interactions seem to strongly influence both models. This further validates the rich chemical and local environment information provided by molecular fingerprints and heuristic methods, which have been used for computational materials science ML^72^ and electrolyte screening efforts,^73^ but not in tandem and for ion transport in intercalation electrodes. The maximum site volume difference, defined as (*V*_*max*_ – *V*_*min*_)/*V*_*min*_ where *V* is the volume enclosed by an atom’s corresponding Voronoi polyhedron,^68,74^ and the coordination difference of Ca^2+^ at the metastable vs transition state site are also identified as important descriptors in the RF and XGB models, respectively. It should be noted that 7 of the 9 most promising candidates exhibit 1D migration pathways (Table 2), as do structural families such as the zircons which exhibit low barriers.^26,27,63^ Aside from the 1D channels in the zircons, the interlocking octahedral motif is identified between layers in NbS_3_. This motif possibly correlates with lower dimensional migration pathways due to local environments that allow for significant nearest-neighbor overlap between interstitial sites (e.g., channels produced by edge or face-sharing polyhedra vs corner-sharing polyhedra).

**Figure 7:**
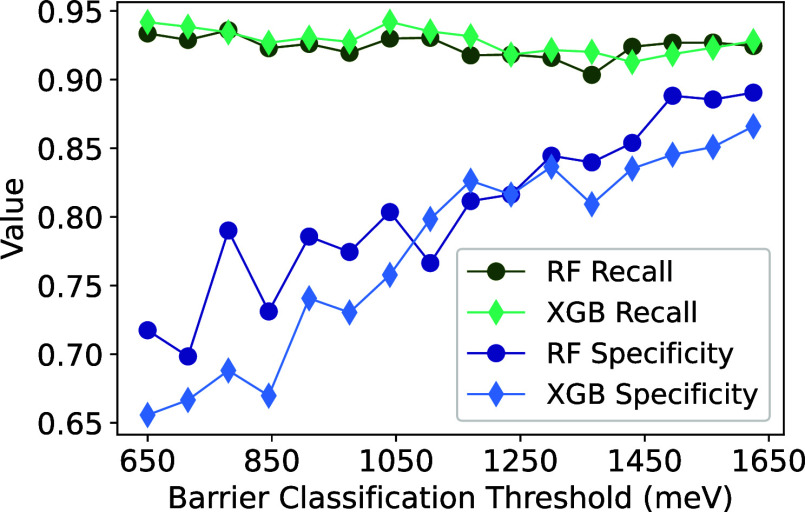
Recall and specificity values for RF and XGB models with varying migration barrier classification thresholds, which are generated from unrelaxed ApproxNEB pathways.

To predict the classification of energy barriers without performing DFT, another set of RF and XGB models is trained and evaluated with feature data from input ApproxNEB structures, which represent unrelaxed ApproxNEB pathways. Since each pathway is initialized with the charge density using the PathFinder algorithm as implemented in Rong et al.’s work,^35^ these unrelaxed structures are decent “guesses" of the minimum energy pathway and yield useful information. However, the most important features from these ML models have unreliable physical meaning since the ApproxNEB structures are not relaxed with DFT. Both the RF and XGB models are generated with varying barrier cutoffs in increments of 10% from 650 meV to classify pathways as low or high energy. Their performance is determined based on their recall and specificity to identify as many low barrier pathways and discard the most high barrier pathways for screening purposes. A threshold greater than 650 meV is acceptable, because it is expected that ApproxNEB overestimates the migration energy with its fewer degrees of freedom during relaxation.^35^ However, maintaining a threshold relatively close to 650 meV is important to identify materials with low enough ion migration energies sufficient to support good ionic transport. As the barrier classification threshold increases, the recall slightly decreases while the specificity rapidly approaches 1 in both models (Figure 7). Depending on the cutoff, one model may outperform the other. For instance, when classifying barriers below 780 meV as acceptable, the RF model is more equipped to correctly identify low barriers and screen out poor candidates with a recall of 0.94 and specificity of 0.79 (compared to 0.93 and 0.69, respectively, with XGB). However, with higher thresholds such as 1,170 meV, the XGB model surpasses the RF model with a recall of 0.93 and specificity of 0.83 (compared to 0.92 and 0.81, respectively, with RF). The rate at which the specificity improves declines beyond 1,170 meV, which is nearly double the barrier cutoff for viable experimental testing. Both models perform similarly with this classification threshold and exhibit the recall values of 0.92–0.93 and specificity values of 0.81–0.83.

We present models for high-throughput ion mobility screening, which is the most time and resource-intensive step in the cathode discovery pipeline. To demonstrate the applicability of these models, the entire screening pipeline is applied to the 2024 version of the Materials Project database, which has an additional 26,900 inorganic crystalline materials compared to the database from 2022. The same pipeline criteria are applied, except for a stricter energy above the hull cutoff of 0.1 eV/atom rather than 0.2 eV/atom for the host material. After discarding candidates that do not have a redox-active metal suitable for Ca intercalation, are neither oxides nor sulfides, contain radioactive elements and/or extractable ions, and lack an experimental ICSD tag, 2,124 materials remain. Candidates that are confirmed to have high electrode cost function values and/or complete ApproxNEB data from the 2022 pipeline are deprioritized. The pretrained graph neural network-based force-field CHGNet^65^ is applied to quickly evaluate the electrode properties of the 758 previously unevaluated candidates in lieu of DFT. Topotactic ion insertion is achieved in 642 materials, and 106 candidates with tier 1 electrode properties are identified. For these 106 materials, migration graphs are created to identify percolating pathways, and minimum energy pathways are initialized with the charge density. Feature data is generated with the resulting input image structures on a local machine, and classification predictions are obtained in less than an hour for these 106 novel candidates. Both the RF and XGB models are applied to classify barriers with a cutoff of 1,170 meV. Our models successfully highlight 18 novel cathode candidates with a high probability of percolating low barrier pathways (Table 3). Notably, the models highlight NASICON-structured Ti_4_Mn(PO_4_)_6_, which has not previously been evaluated as a cathode material. Although monazite SrCrO_4_ has similar electrode properties as tetragonal zircon ABO_4_ chromates (e.g., intercalation voltage, volume change, gravimetric capacity), SrCrO_4_ has a lower conversion voltage and much better phase stability for the same level of Ca intercalation in the zircons.^27^ Tavorite VPO_4_F has been explored as a high voltage Li and K cathode,^75^ and it presents itself as a high capacity Ca cathode candidate with a decent voltage of 3.4 V. High Li conduction has been confirmed in scheelite oxides,^76^ which supports our findings for good Ca-ion mobility in Ce_2_(WO_4_)_3_. These results suggest that the classification models can quickly detect low barrier pathways with decent accuracy and accelerate the screening pipeline. Structural similarities are found between Ba_2_CoO_4_ and Ba_2_FeO_4_, resulting in a total of 17 unique structure types. Although performing ApproxNEB or NEB can more reliably validate the barriers for these materials from the 2024 database, there are additional indicators of low migration barriers based on the following characteristics: unfavorable coordination for Ca^2+^ in the metastable site and preferred coordination in the transition state site (e.g., coordination of 2 and 6, respectively, for Ba_2_FeO_4_), maximum Ca coordination change of 1–2 in 33% of the candidates, consistently low overlap motif (e.g., overlap of 1–2 in VPO_4_F, SbSO_2_F_7_, SbS(BrF_2_)_3_, SrCrO_4_, V_2_Co(PO_5_)_2_), and tetrahedral overlap motif (e.g., W(ClO)_2_). Interestingly, 73% of the oxides have a low volume per anion whereas a majority of the sulfides have a high volume per anion, which tends to correlate with better screening and mobility.

**Table 3:** Electrode Properties for Novel Candidates with ML-Predicted, Low Barrier, Percolating Pathways from the 2024 Materials Project Database^a^^b^

Composition MP-ID	Prototype Structure	Intercalation Voltage (V)	Charged Stability (meV/Atom)	Discharged Stability (meV/Atom)	ΔVolume (%)	Gravimetric Capacity (mA h/g)
Ca0–0.25MoPCl_8_O	-	2.9	19	21	0	31
mp-560214						
Ca0–0.5Co(ReO_4_)_2_	-	2.3	25	12	2	46
mp-550479						
Ca0–0.25Fe_3_Te_3_ClO_10_	-	2.7	39	40	3	18
mp-1200464						
Ca0–0.25EuFMoO_4_	-	3.4	0	10	6	39
mp-582540						
Ca0–0.5SbSCl_9_	-	3.1	7	36	0	54
mp-557809						
Ca0–0.25SbS(BrF_2_)_3_	-	3.6	6	9	8	26
mp-555288						
Ca0–1Ti_4_Mn(PO_4_)_6_	NASICON	2.1	27	38	3	63
mp-19383						
Ca0–0.25Ba_2_FeO_4_	-	2.3	1	18	11	33
mp-542120						
Ca0–0.25SrCrO_4_	monazite	2.4	56	69	5	63
mp-510607						
Ca0–0.25Ba_2_CoO_4_	Ba_2_TiO_4_	2.0	42	48	9	33
mp-17478						
Ca0–0.5VPO_4_F	tavorite	3.4	15	47	12	145
mp-1104878						
Ca0–0.5W(ClO)_2_	-	2.7	87	82	3	87
mp-27937						
Ca0–0.5Nb_4_NiS_8_	-	1.6	1	13	10	38
mp-1192540						
Ca0–0.5Nb_4_Te_9_I_4_O	-	1.7	21	41	1	13
mp-558408						
Ca0–0.5V_2_Co(PO_5_)_2_	lazulite	2.7	47	76	6	67
mp-548753						
Ca0–0.5Tl_3_SbS_4_	-	1.7	4	54	8	30
mp-8378						
Ca0–0.25SbSO_2_F_7_	-	3.7	44	64	3	41
mp-1197121						
Ca0–0.5Ce_2_(WO_4_)_3_	scheelite	2.0	77	100	0	26
mp-17686						

a Voltages are with respect to Ca/Ca^2+^.

b The electrodes are displayed in order of lowest to highest total cost function value, with the most favorable candidate listed first.

## Conclusions

A high-throughput computational pipeline is built to discover novel Ca cathode candidates and structural motifs that support good ion mobility. Cost functions are developed to methodically rank materials by their electrode properties and are shown to effectively prioritize cathodes for minimum energy pathway evaluation. ApproxNEB is performed for 242 systems, resulting in the largest multivalent-ion mobility data set to our knowledge, consisting of 5,508 relaxed supercell structures across 182 structure groups and 48 elements. A promising candidate with a low NEB barrier of 168 meV is synthesized and experimentally tested, yielding a reversible capacity of 25 mA h/g. While encouraging, this lower than expected capacity may be attributed to the 1D migration topology of the material, suggesting that nanosizing could improve its performance. With this migration data, material design rules that govern Ca-ion mobility are further elucidated. Notably, the tetrahedral overlap is frequently present in pathways with low barriers, and the interlocking octahedral motif is identified in NbS_3_, which exhibits remarkable ion mobility with several working ions similar to the zircons.^26,27,63^ It may be of interest to the scientific community to extend the search for systems with the tetrahedral overlap in potential migration pathways and evaluate barriers with other mobile species in these structural families. Relevant features are generated to produce ML models for ion mobility screening. Trained on ApproxNEB data for 612 symmetrically unique pathways across 213 materials, the reported RF and XGB models successfully predict barrier classifications with an accuracy of 92%. We find the AGNI fingerprint^70^ and bond valence site energy^71^ features to heavily influence the models as the most relevant chemical and structural descriptors for mobility analysis. To demonstrate their effectiveness, the models are applied to the 2024 version of the Materials Project database, excluding the materials previously examined, in an accelerated search for novel Ca intercalation host materials using ML force-fields. As a result, 18 new candidate Ca cathode candidates with the potential for good Ca-ion mobility are identified.

In summary, a total of 27 novel and promising Ca cathodes are flagged across the 2022 and 2024 iterations of the screening pipeline, which may warrant further analysis with DFT and experimental validation. It may be of interest to evaluate the electrode properties of the highlighted candidates with higher levels of Ca intercalation and apply ML force-fields to quickly gauge their potential as cathode materials. Although the constructed RF and XGB models are useful screening tools for ion mobility with peak time and resource efficiency, they may incorrectly classify up to 8% of actual low barrier pathways. To improve these models, we stress the importance of highly curated, systematic data sets and validation to ensure robust training. We also propose the use of ML force-fields to generate image structures that are closer to the true minimum energy pathway, rather than relying on feature values from structures initialized with the charge density. Furthermore, since 78% of the best candidates with confirmed low barriers exhibit percolating 1D diffusion pathways, it is suggested to employ screening of percolation topology to better inform macroscopic ionic conductivity. The high-throughput screening tools discussed above may be leveraged in other applications, especially those with similar criteria for ion mobility (e.g., conductors), to accelerate the search for high-performance materials.

## Supporting Information Available

Cost function graphs for electrode properties; demonstration of the tiered electrode ranking process; Ca_*x*_W_2_O_3_(PO_4_)_2_ phase stability data and electrode properties; Ca_*x*_W_2_O_3_(PO_4_)_2_ NEB vs AIMD pathway comparison; Ca_*x*_W_2_O_3_(PO_4_)_2_ AIMD Arrhenius plot; W_2_O_3_(PO_4_)_2_ XRD pattern; [Ca, Li, Mg]_*x*_NbS_3_ phase stability data and electrode properties; ApproxNEB barrier vs volume per anion data; nearest-neighbor overlap and site energy difference data for ApproxNEB image structures; ApproxNEB barrier distribution; AGNI fingerprint differences between pathways (PDF)
