# Supplementary material for: Evaluating Material Design Principles for Calcium-Ion Mobility in Intercalation Cathodes
Source: Chem Mater. 2024 Dec 30;37(1):507–19. doi: 10.1021/acs.chemmater.4c02927 (PMC11736685; doi:10.1021/acs.chemmater.4c02927)
Supplement: Supplementary file 1 — cm4c02927_si_001.pdf [file cm4c02927_si_001.pdf]

Supporting Information: Evaluating Material Design Principles for Calcium-Ion Mobility in Intercalation Cathodes

Jiyoon Kim,[†,‡] Dogancan Sari,[†,‡] Qian Chen,[‡] Gerbrand Ceder,[†,‡] and Kristin A. Persson[*,†,‡]

†*Department of Materials Science and Engineering, University of California, Berkeley, California 94704, United States*

‡*Materials Sciences Division, Lawrence Berkeley National Laboratory, Berkeley, California 94720, United States*

E-mail: kapersson@lbl.gov

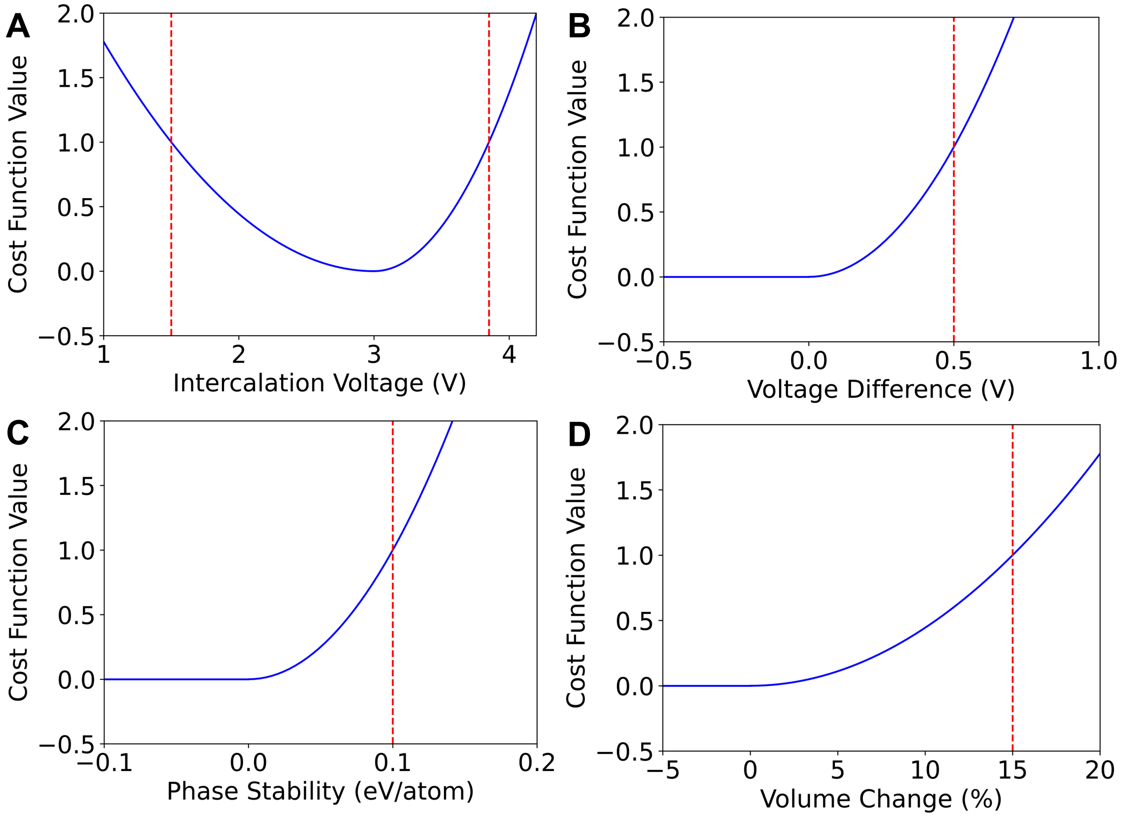

Figure S1: Cost functions to rank electrodes. (A) Intercalation voltage with respect to Ca/Ca^{2+}. (B) Conversion voltage minus the intercalation voltage with respect to Ca/Ca^{2+}. (C) Energy above the hull value of the host (charged) and calciated (discharged) structure. (D) Change in volume upon intercalation.

Table S1: Application of cost functions on electrode properties to rank cathode candidates. The three tiers of candidates are classified from top to bottom as such: promising, not ideal, and not viable for experimental consideration. Voltages are with respect to Ca/Ca^{2+}. The charged and discharged stability describe the energy above hull values for the host and calciated structures, respectively.

Composition MP-ID	Total Cost Function Value	Intercalation Voltage (V)	Conversion Voltage (V)	Charged Stability (meV/atom)	Discharged Stability (meV/atom)	Δ Volume (%)
$Ca_{0-0.5}Ba_5Re_5O_{16}$ mp-698206	0.03	3.1	2.9	8	0	2
$Ca_{0-0.5}V_2Ni(PO_5)_2$ mp-554784	1.00	2.8	3.2	24	48	4
$Ca_{0-0.25}MnPbO_3$ mp-1106337	2.86	2.9	3.2	83	92	13
$Ca_{0-0.5}Mo_8O_{23}$ mp-2669	1.10	2.7	3.2	5	21	1
$Ca_{0-0.5}Ti(PS_3)_2$ mp-13666	2.58	1.5	2.1	0	59	1
$Ca_{0-0.5}Ti_3FeS_6$ mp-16335	5.74	1.3	1.9	91	127	11
$Ca_{0-0.25}Ba_3FeO_5$ mp-30093	5.74	4.4	2.6	156	50	6
$Ca_{0-0.5}Sm_3FeO_6$ mp-1105484	12.92	0.5	1.9	0	129	14
$Ca_{0-1}CuO$ mp-14549	437.35	0.1	2.5	167	1633	178

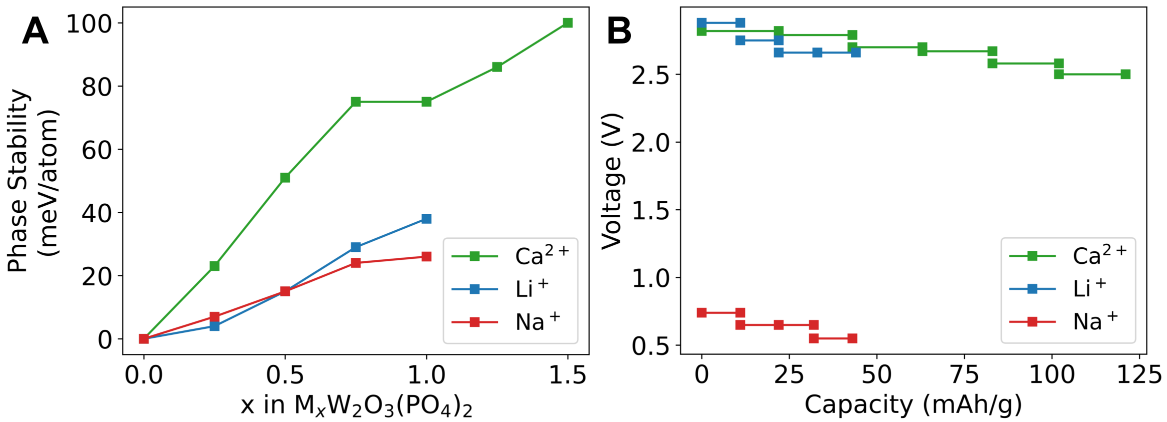

Figure S2: (A) Phase stability of $W_2O_3(PO_4)_2$ intercalated with Ca^{2+}. (B) Voltage values that correspond to intercalation levels shown in Figure A.

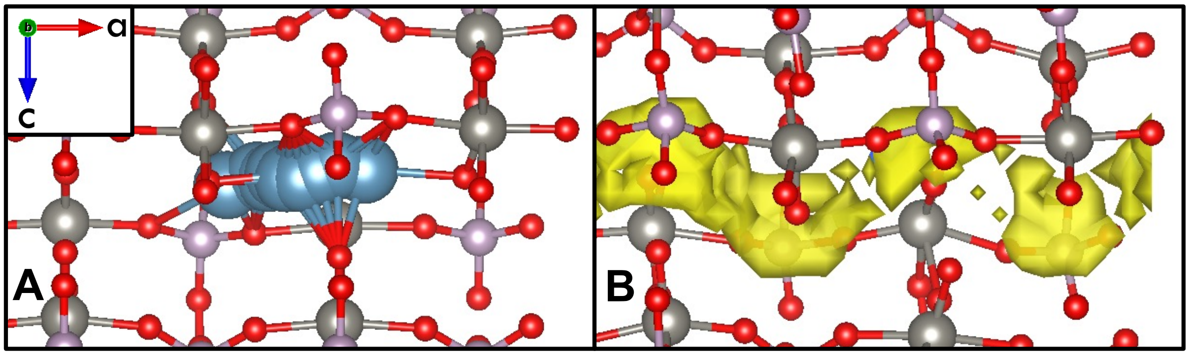

Figure S3: (A) Migration pathway of Ca^{2+} from NEB calculations. (B) Migration pathway of Ca^{2+} from AIMD calculations at 900 K.

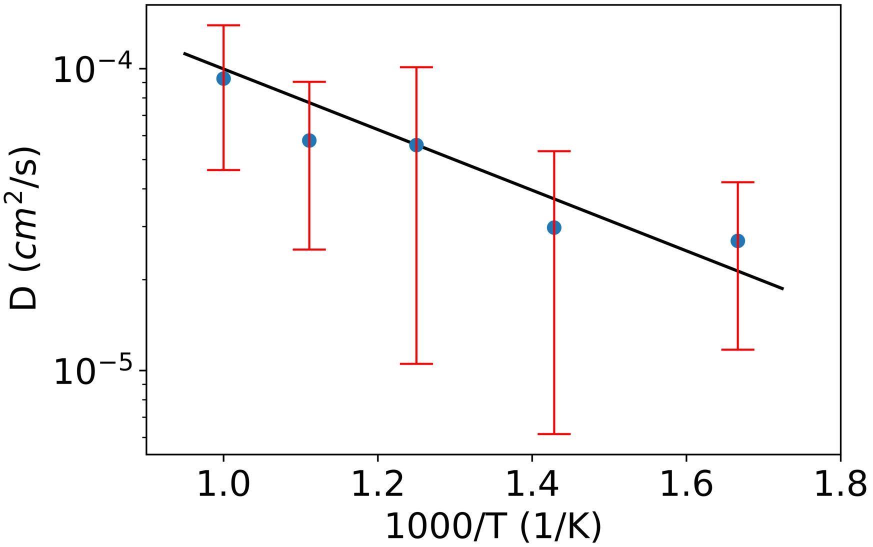

Figure S4: Arrhenius plot of Ca diffusivity in $Ca_xW_2O_3(PO_4)_2$ in the dilute lattice limit (x $\leq$ 0.125 with 1 Ca-ion per supercell structure) based on AIMD simulations.

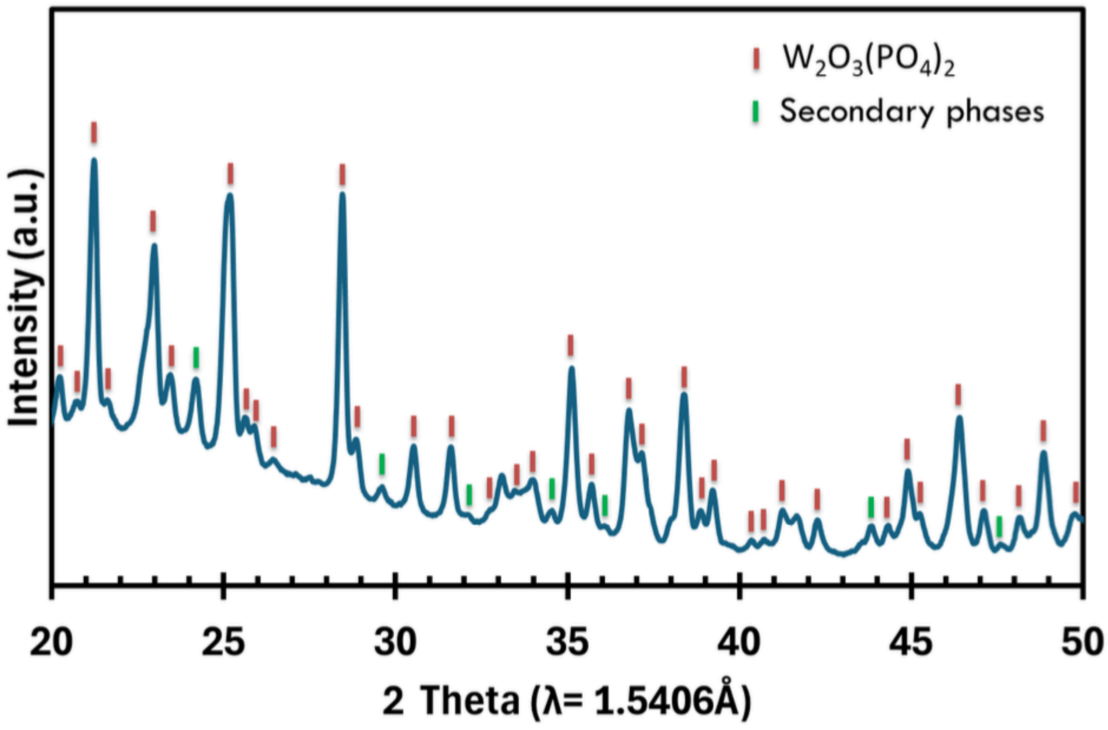

Figure S5: XRD pattern of $W_2O_3(PO_4)_2$.

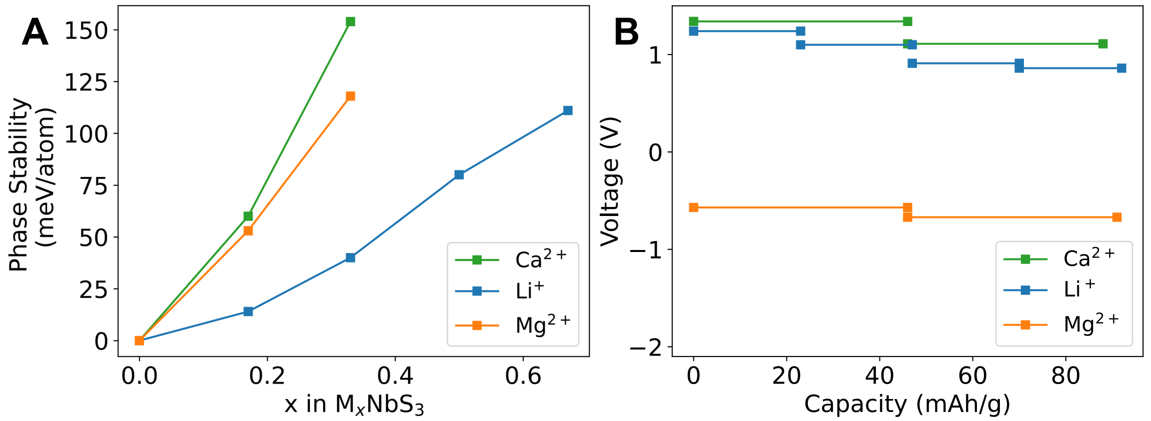

Figure S6: (A) Phase stability of NbS_3 intercalated with Ca^{2+}, Li^+, and Mg^{2+}. M represents the working ion. (B) Voltage values that correspond to intercalation levels shown in Figure A.

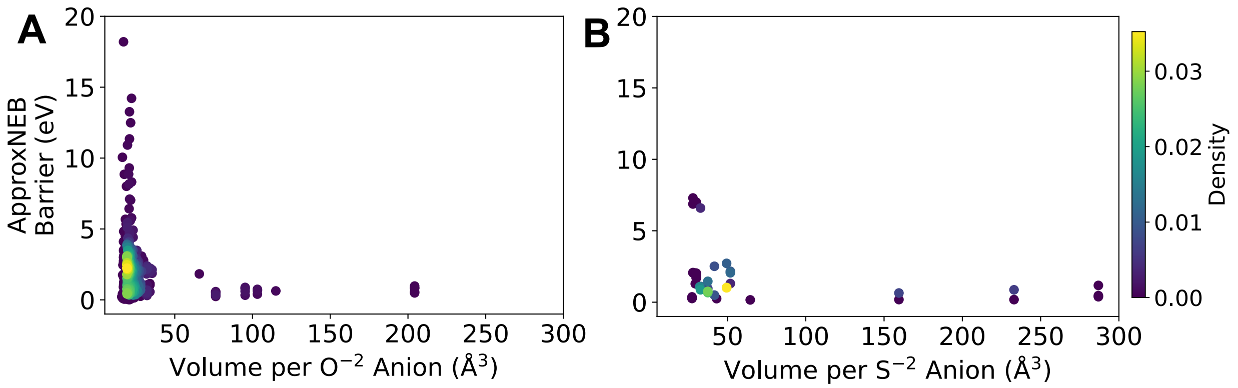

Figure S7: ApproxNEB barrier vs. volume per (A) O^{2-} anion and (B) S^{2-} anion. The density of the data is relative to each anion dataset.

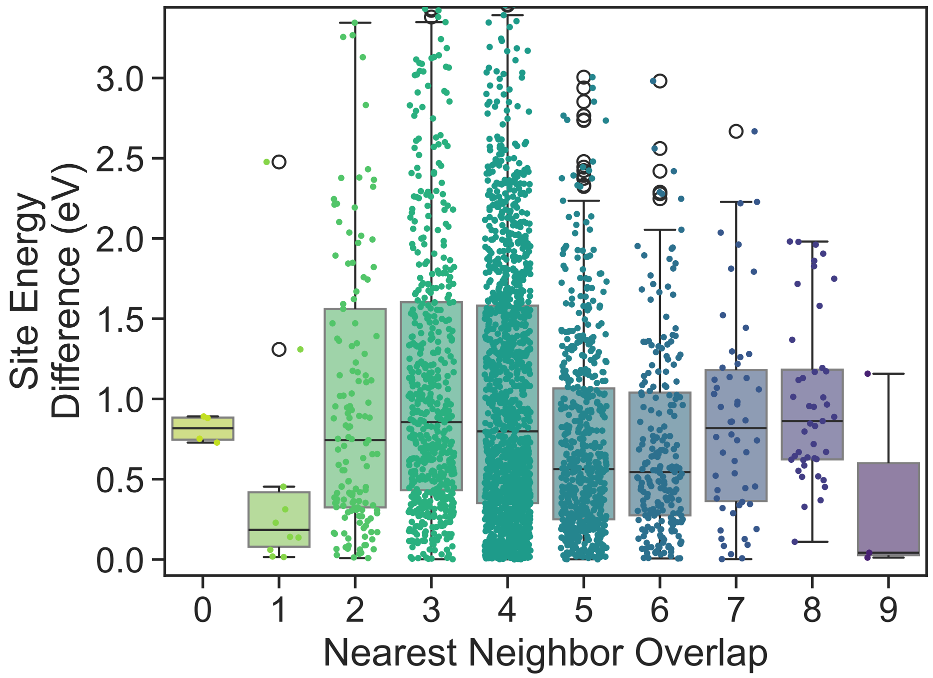

Figure S8: Nearest-neighbor overlap and respective site energy difference of ApproxNEB image structures. Outlier data with site energy differences > 3.5 eV are not shown.

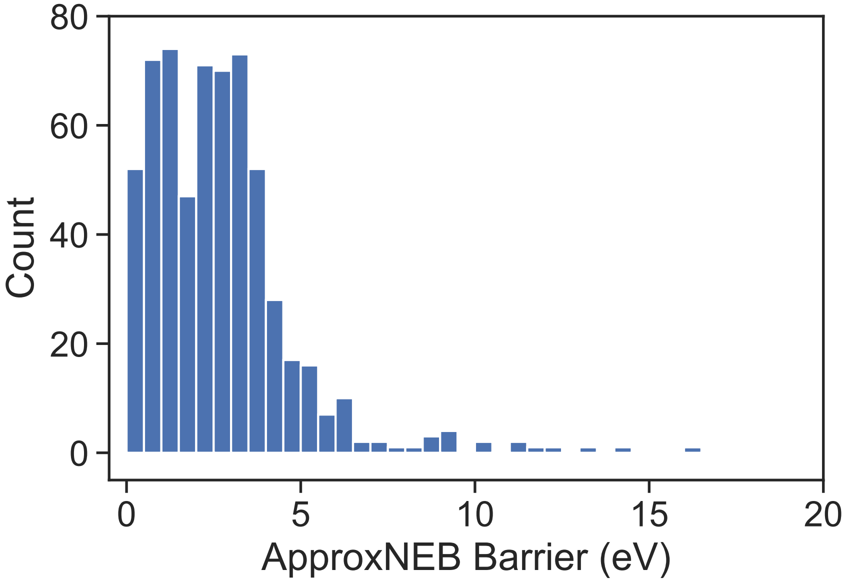

Figure S9: ApproxNEB barrier distribution for 612 relaxed minimum energy pathways.

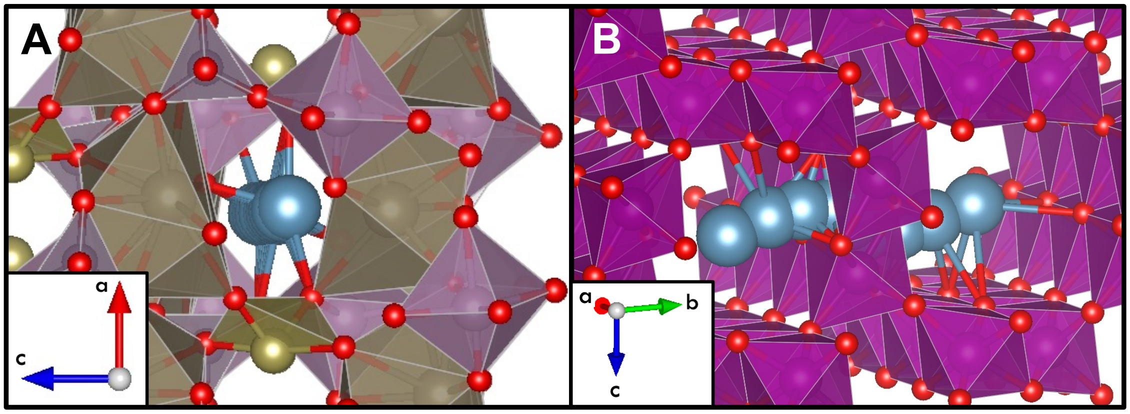

Figure S10: Migration pathway of Ca^{2+} (in blue) in (A) $Tl_2TeMo_2(PO_7)_2$ with a barrier of 156 meV and (B) MnO_2 with a barrier of 9,211 meV.
